# Eukaryotic Communities in Aquatic Mesocosms with and without Calcined Dolomite Investigated Using 18S rRNA Amplicon-Based Metagenomics

**DOI:** 10.1128/MRA.01025-21

**Published:** 2021-12-02

**Authors:** Yuki Itaya, Shogo Nakamura, Tatsushi Akiyama, Kenji Kuninishi

**Affiliations:** a Cement/Concrete Research Laboratory, Sumitomo Osaka Cement Co., Ltd., Chiba, Japan; University of California, Riverside

## Abstract

We report the 18S rRNA gene amplicon data from aquatic mesocosms with and without calcined dolomite. Intramacronucleata and Eumetazoa were present in roughly the same amounts in the water phase in both mesocosms. Chlorophyceae and several groups were detected as the major eukaryotes in the microbes attached to the calcined dolomite surface.

## ANNOUNCEMENT

The discharge of wastewater, which contains a large amount of nitrogen and phosphorus, into rivers may destroy their ecosystems. Calcined dolomite (c. d.) is known to have a high sorption capacity for phosphorus and is used as an absorbent. However, the effect of the calcined dolomite on river ecosystems remains unknown. Therefore, we constructed an aquatic mesocosm in a laboratory using river water and performed 18S rRNA gene amplicon sequencing.

In May 2020, sediments and water were collected from the Shiba River, Saitama Prefecture, Japan (35.816°N, 139.729°E). Microbes in the river water were collected by filtration (HAWP04700; Millipore). Two mesocosms were set up. Each mesocosm consisted of a polypropylene water tank, 1 L sediment, and 2 L river water. For one mesocosm, 40 g calcined dolomite was laid in a hanging mesh basket that was not in contact with the sediment. The calcined dolomite was prepared by dolomite calcination (800°C for 20 min). The particle size distribution of the calcined dolomite was 3 to 7 mm. The mesocosm was kept at 20°C and 20,000 lx under a light/dark ratio of 12 h:12 h. The other mesocosm was kept under the same conditions as above, without the addition of calcined dolomite for comparison.

Sampling was conducted on day 47 from the start of incubation. Microbes in the water phase were collected by filtration, the same as for river water. Microbes attached to the calcined dolomite were detached by sonication and gathered by filtration.

DNA was extracted from the membrane filters using the Extrap soil DNA kit Plus v2 (Nippon Steel Eco-Tech Co., Ltd., Tokyo, Japan). The V4 region of the 18S rRNA gene was amplified with TAReuk454FWD1 (1) and TAReukREV3 (2) using the KAPA 3G plant PCR kit (Kapa Biosystems). The amplification profile consisted of 3 min at 95°C, 30 cycles of 20 s at 95°C, 15 s at 53°C and 60 s at 72°C, followed by a final extension of 5 min at 72°C. The sequencing library was prepared using the Illumina Nextera XT index kit v2. Sequencing of the 18S rRNA gene was performed using the Illumina MiSeq v3 reagent kit (2 × 300-bp paired-end method) on the Illumina MiSeq platform. The DADA2 plugin (3) in QIIME2 v2019.10 was applied to quality filter, denoise, and merge the paired-end reads, and remove chimeric sequences. The QIIME2 naive Bayesian classifier was used for taxonomic classification against the SILVA 18S rRNA database v132 (4). Default parameters were used for all software contained in the QIIME2 environment.

**FIG 1:**
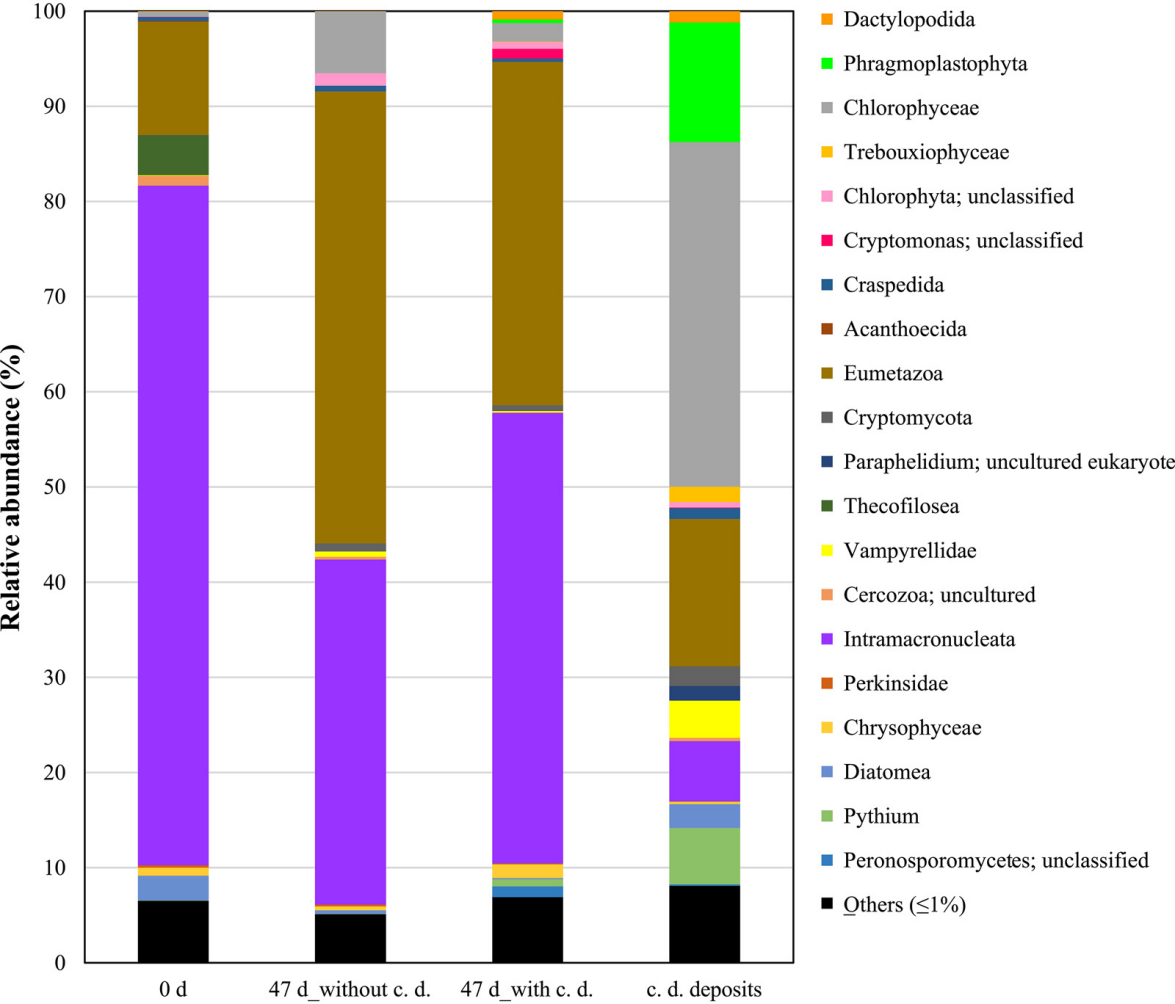
Eukaryotic community structures based on an 18S rRNA gene sequence analysis. The eukaryotic organism groups indicated in this figure are classified at level 4 in the SILVA 18S rRNA database. Abbreviations: 0 d, environment water on day 0; 47 d without c. d., water in the aquatic mesocosms without calcined dolomite on day 47; 47 d with c. d., water in the aquatic mesocosms with calcined dolomite on day 47; c. d. deposits, biofilm on the surface of the calcined dolomite on day 47.

The average merged read length was 380 bp, and a total of 802,355 high-quality reads were obtained (the number of merged reads for each sample was between 155,306 and 269,082). Intramacronucleata was the predominant eukaryotic group in the river water (Fig. 1). The relative abundance of Intramacronucleata and Eumetazoa reached over 80% in the water phase in the mesocosms. Various organisms, such as Chlorophyceae, Phragmoplastophyta, Eumetazoa, Intramacronucleata, Diatomea, and *Pythium*, were detected in the biofilm on the surface of the calcined dolomite (c. d. deposits).

### Data availability.

The 18S rRNA gene amplicon sequence data sets have been deposited in the DDBJ GenBank under the SRA accession numbers DRR316761, DRR316762, DRR316763, and DRR316764 and the BioProject accession number PRJDB12231.
